# Achieving excellent superplasticity of Mg-7Zn-5Gd-0.6Zr alloy at low temperature regime

**DOI:** 10.1038/s41598-018-38420-7

**Published:** 2019-03-13

**Authors:** Siqi Yin, Zhiqiang Zhang, Jiamin Yu, Zilong Zhao, Min Liu, Lei Bao, Zheng Jia, Jianzhong Cui, Ping Wang

**Affiliations:** 1 Key Lab of Electromagnetic Processing of Materials, Ministry of Education, Northeastern University, 314 Mailbox, Shenyang, 110819 China; 2 College of Materials Science and Engineering, Northeastern University, Shenyang, 110819 China; 3 School of Materials, University of Manchester, Sackville Street, Manchester, M13 9PL UK; 4 School of Civil and Environmental Engineering, Nanyang Technological University, 50 Nanyang Avenue, 639798 Singapore, Singapore; 5 College of Mechanical Engineering, Shenyang University, Shenyang, 110044 China

## Abstract

Mg-7Zn-5Gd-0.6Zr (wt%) alloy strengthened with quasicrystal phase (I-Mg_3_Zn_6_Gd phase) is prepared through hot extrusion and subsequent heat treatments. The low temperature (range from 25 °C to 250 °C) superplastic deformation behavior of the as-extruded, aging treated (T5) and solution and aging treated (T6) alloys are investigated. The results reveal that a superior superplastic elongation of 863% is obtained at 250 °C and strain rate of 1.67 × 10^−3^ s^−1^ and the elongation of this alloy increases with the increasing tensile temperature. Detailed microstructural analyses show that I-Mg_3_Zn_6_Gd phase and W-Mg_3_Gd_2_Zn_3_ phase are crushed into small particles during extrusion. A high density of nanoscale I-phase precipitates after T5 treatment. Dynamic recrystallization occurs in as-extruded Mg-7Zn-5Gd-0.6Zr alloy. The T5-treated Mg-7Zn-5Gd-0.6Zr alloy shows a relatively weak basal texture intensity, a large number fraction of high angle boundaries and a very finer grain structure (3.01 μm). During superplastic deformation, the nanoscale I-phase is slightly elongated and the microstructure is still equiaxed grains. The superplastic mechanism of the alloy is grain boundary sliding (GBS) accommodated by dislocation movement and static recrystallization. The cavity nucleation at the nanoscale I-phase/α-Mg matrix boundaries or grain boundaries and the cavity stringer formation leads to final fracture.

## Introduction

Magnesium alloys are the lightest structural materials in the earth which have received considerable attention for applications leading to fuel efficiency and green environment^1–3^. However, the utilization of the complex structural components in aerospace, aviation and automotive industries is limited because of their bad formability at middle and low temperatures and inferior strength and ductility balance. Among this alloy family, Mg-Zn-Gd (RE) series have been a subject of intensive studies due to the considerable room-temperature ductility, thermal stability^4,5^ and formation of stable icosahedral quasicrystal structure I-phase (Mg_3_Zn_6_Gd) to overcome these disadvantages of the alloy^6–8^. Mg-Gd-Zn alloys with icosahedral quasicrystal (I-phase) have been accustomed choices for their excellent mechanical properties, such as high hardness, stability and low interface energy with matrix. Miao *et al*.^9^ recently identified the as-extruded I-phase (Mg_3_Zn_6_Gd) containing Mg-2.4Zn-0.8Gd (wt%) alloy showed a tensile strength of 338 MPa and large elongation of 24.1% simultaneously. Mg-1.5Zn-0.25Gd (at%) alloy fabricated through extrusion and compression compound techniques exhibited the moderate yield strength of 161 MPa and excellent elongation of 31.4%^10^. Moreover, it is noted that the Mg-Zn-RE alloys with quasicrystal I-phase have also been developed for applications at elevated temperatures because of the high eutectic temperature and a coherent quasicrystal I-phase/α-Mg phase interface, even at higher temperatures^11,12^. For instance, Bae *et al*.^13^ investigated that the extruded and hot-rolled Mg-1.7Y-7.6Zn-1.8Zr (wt%) alloy established a maximum elongation of 780% at 450 °C and the strain rate of 5 × 10^−4^ s^−1^. Yang *et al*.^12^ reported that the Mg-1.2Y-7.12Zn-0.84Zr (wt%) alloy showed a maximum elongation of 1110% at 450 °C and a high strain rate of 1.0 × 10^−2^ s^−1^ which was hot-rolled and friction stir processed (FSP). The above-mentioned consequences reveal that the optimal superplastic temperatures are restrained to high temperatures above 300 °C or complex severe deformation mechanism. It is also noteworthy that when comparing with Y element, Gd displays a higher solid solubility in magnesium alloys. Solid solution strengthening and precipitation hardening are simultaneously obtained due to the Gd addition and thermal stability of the microstructures could also be strengthened later^14,15^. The diluted Gd-containing Mg-Zn-Gd-Zr alloys with low-temperature superplastic forming are beneficial to energy reductions, shortening the production period and lower the cost in industries.

Superplastic materials acquire through strict conditions with rigorous temperatures control and definite strain rates. The homogeneous and fine-grained alloys with high angle grain boundaries (HAGB)^16^ could often achieve superplasticity. It is possible to utilize severe plastic deformation (SPD), such as equal channel angular pressing (ECAP)^17^, high-pressure torsion (HPT)^18^, multidirectional forging (MDF)^19,20^ and friction stir processing (FSP)^21^ to achieve aforesaid microstructures in magnesium alloys. Previously, superplastic deformation has been obtained through severe plastic deformation (SPD) techniques, but the cost is high and the time is also consuming. Therefore, it is quite urgent to achieve ultra-fine grains through proper alloying elements and conventional continuous processing technologies such as extrusion or rolling. Thus, the Mg-5Gd-7Zn-0.6Zr (wt%) alloy containing I-Mg_3_Zn_6_Gd phase is manufactured by traditional casting and indirect extrusion to investigate the superplastic behavior at temperatures less than 250 °C through the microstructural characterization, cracking behavior and texture evolution. This is important for processing technology and alloy component design, so as to fabricate lower cost magnesium alloys.

## Experimental Procedure

The material used was Mg-7Zn-5Gd-0.6Zr (wt%) alloy with a practical composition of Mg-6.87Zn-4.91Gd-0.42Zr (wt%). The chemical compositions were determined by inductively coupled plasma (ICP) analysis. To prepare the experimental alloy, pure Mg (99.95 wt%), pure Zn (99.9 wt%), Mg-25Gd (wt%) and Mg-30Zr (wt%) intermediate alloys were used, melting and casting details have been depicted elsewhere^7^. The initial cast ingots were homogenization treated at 430 °C for 12 h and then quenched in water. Indirect extrusion was then carried out at 400 °C, the extrusion ratio was 15 and the ram speed was 3.7 mm/s. Some extruded rods were subjected to aging treatment at 200 °C with aging time from 2 h to 117 h and peak-aged sample was obtained at 16 h (T5). The other extruded specimens were solution treated at 430 °C for 8 h before peak-aging treatment (T6). Finally, all the specimens were quenched in water. The final as-extruded, peak-aged and solution + peak-aged samples were denoted as E, E + T5 and E + T6.

According to the ASTM (B557M-10) standard, tensile specimens have the original gauge length of 35 mm and diameter of 6 mm. The samples were cut from the extruded rods along the hot-extruded direction. Tensile tests were conducted at 25 °C, 150 °C, 200 °C and 250 °C at an initial strain rate of 1.67 × 10^−3^ s^−1^ using a Shimadzu AG-X (100 kN) machine. All the specimens were held at the desirable testing temperature for 5 min prior to tensile testing to reach the thermal equilibrium. At least three specimens were used to obtain consistent tensile properties.

**Figure 1:**
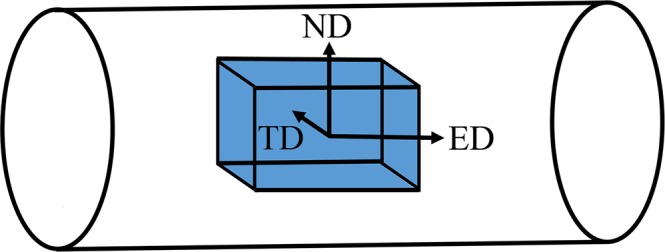
Schematic diagram of the direction definition of extruded rods.

The constituent phases of E, E + T5 and E + T6 samples were detected through X-ray diffraction (XRD; X′ Pert Pro MPD), and the scanning angle was from 10° to 90° with a speed of 8°/min. Microstructures were examined by optical microscope (OM; OLYMPUS PMG 3) and scanning electron microscope (SEM; Zeiss ULTRA 55), the microstructures were taken from longitudinal sections of the extruded rod. A transmission electron microscope (TEM; JEM-ARM200F) was used to characterize the detailed microstructures of the alloys. Specimens for the XRD testing were grinded with 400-grit to 3000-grit SiC papers, the SEM and OM samples were etched with the ethanol solution of picric acid and glacial acetic acid (5 ml glacial acetic acid, 2.5 g picric acid, 25 ml ethanol and 5 ml water) after mechanically polish and milling. The TEM specimens with a diameter of 3 mm were ion-milled with Gatan 695, (5.0 kV ion gun energy under 10° milling angle, subsequently, 3.0 kV ion gun energy under 3.5° milling angle). For electron back scattering diffraction (EBSD) examinations, samples were ground with 5000 grit SiC papers, polished with 0.5 μm diamond compound, and then argon ion polishing for 2 h. The EBSD result was analyzed by software (OIM, HKL-Channel 5) and the measured pole figures contained {0002} and {$10\bar{1}0$}. The ED, ND and TD were representative of extrusion direction, normal direction and transverse direction as shown in Fig. 1. The misorientation angles between the adjacent grains were used to identify the high angular grain boundary (HAGB, θ ≥ 15°) and low angular grain boundary (LAGB, 2° ≤ θ ≤ 15°), as indicated by black and green lines, respectively. Linear intercept method through at least ten areas for each alloy (with the software of Image Pro Plus) was analyzed to calculate the grain sizes from the band contrast images.

## Results

### Mechanical behavior

**Figure 2:**
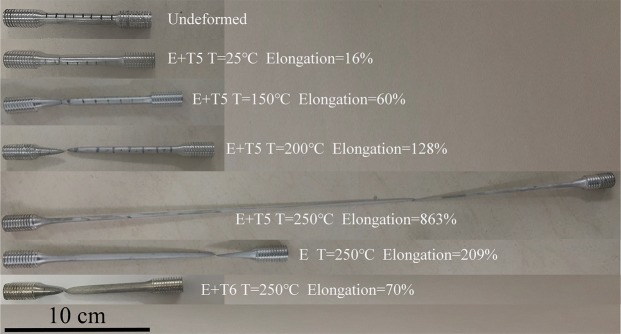
Representative initial and fractured tensile specimens for superplastic deformation of GZ57K alloy.

Original and fractured tensile specimens are displayed in Fig. 2. The optimal elongation of 863% is achieved from the specimen tested at 250 °C and 1.67 × 10^−3^ s^−1^, in which the diffusional necking appears within the uniform gauge length.

**Figure 3:**
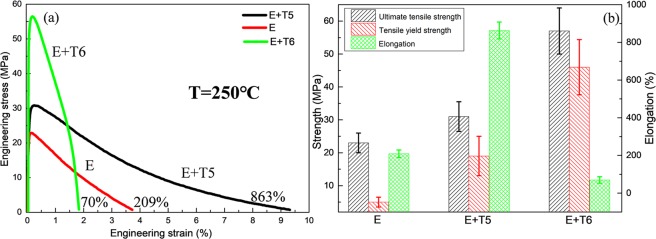
(**a**) Tensile stress-strain curves tested at 250 °C; (**b**) the strength and elongation to failure of the three conditions.

**Figure 4:**
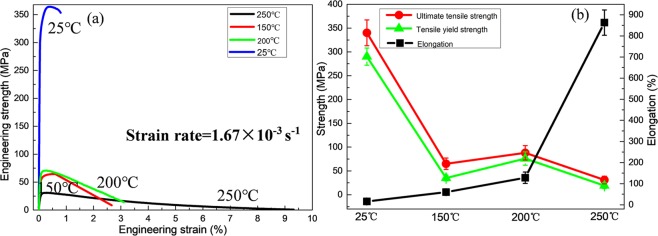
(**a**) Tensile stress-strain curves tested at 1.67 × 10^−3^ s^−1^; (**b**) the strength and elongation to failure in different conditions.

Figure 3 depicts the representative engineering stress-strain curves of the E, E + T5 and E + T6 alloys which are tested at 250 °C. The tensile yield strength, ultimate tensile strength and elongation of the extruded alloy are 5 MPa, 23 MPa and 209%, respectively. What is more important, the elongation increases to 863% after T5 treatment and the strength insignificantly increases to 19 MPa and 31 MPa at the same time. Through T6 treatment, the strength increases to 46 MPa and 57 MPa, but the elongation rapidly decreases to 70%. Figure 4 demonstrates the tensile stress-strain curves of the E + T5 alloys obtained at various temperatures with stable strain rate of 1.67 × 10^−3^ s^−1^. It can be observed from Fig. 4(a) that the alloys exhibit relatively weaker strain hardening without obvious stress peaks above 25 °C. The tensile strength decreases dramatically and the elongation increases tremendously with the increasing temperature. It is worth noted that the elongation is 863% at the strain rate of 1.67 × 10^−3^ s^−1^ under 250 °C test showing that the superplasticity can be achieved in E + T5 alloy at the high strain rate.

### Microstructures before superplastic transformation

**Figure 5:**
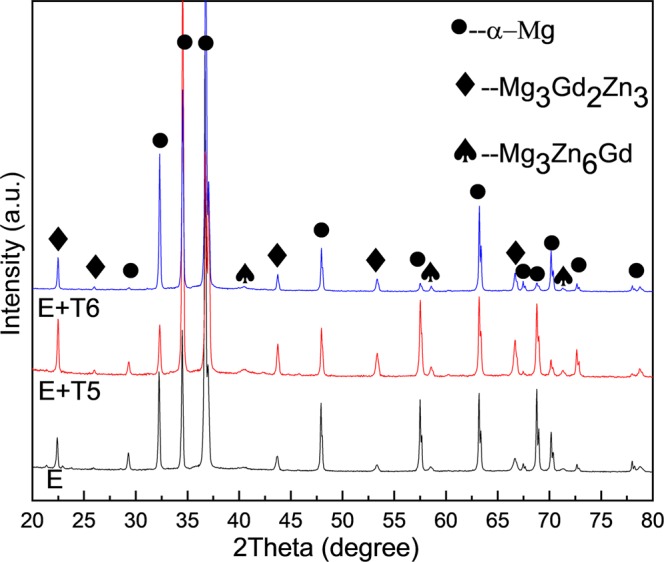
XRD patterns of the GZ57K alloy.

The X-ray diffraction patterns of E, E + T5 and E + T6 alloys are exhibited in Fig. 5. Results confirm that the experimental alloys mainly compose of α-Mg, W-Mg_3_Gd_2_Zn_3_ phase and I-Mg_3_Zn_6_Gd phase. With the solution and aging process, the phase compositions in the three conditions do not vary obviously except for the peak intensity fluctuation. Through T5 treatment, diffraction peaks intensity of W-Mg_3_Gd_2_Zn_3_ phase and I-Mg_3_Zn_6_Gd phase is stronger than that in the as-extruded condition, and diffraction peaks intensity of the second phases in the T6 condition is almost unchanged. Results of diffraction peaks have been manipulated through the normalized procedure.

**Figure 6:**
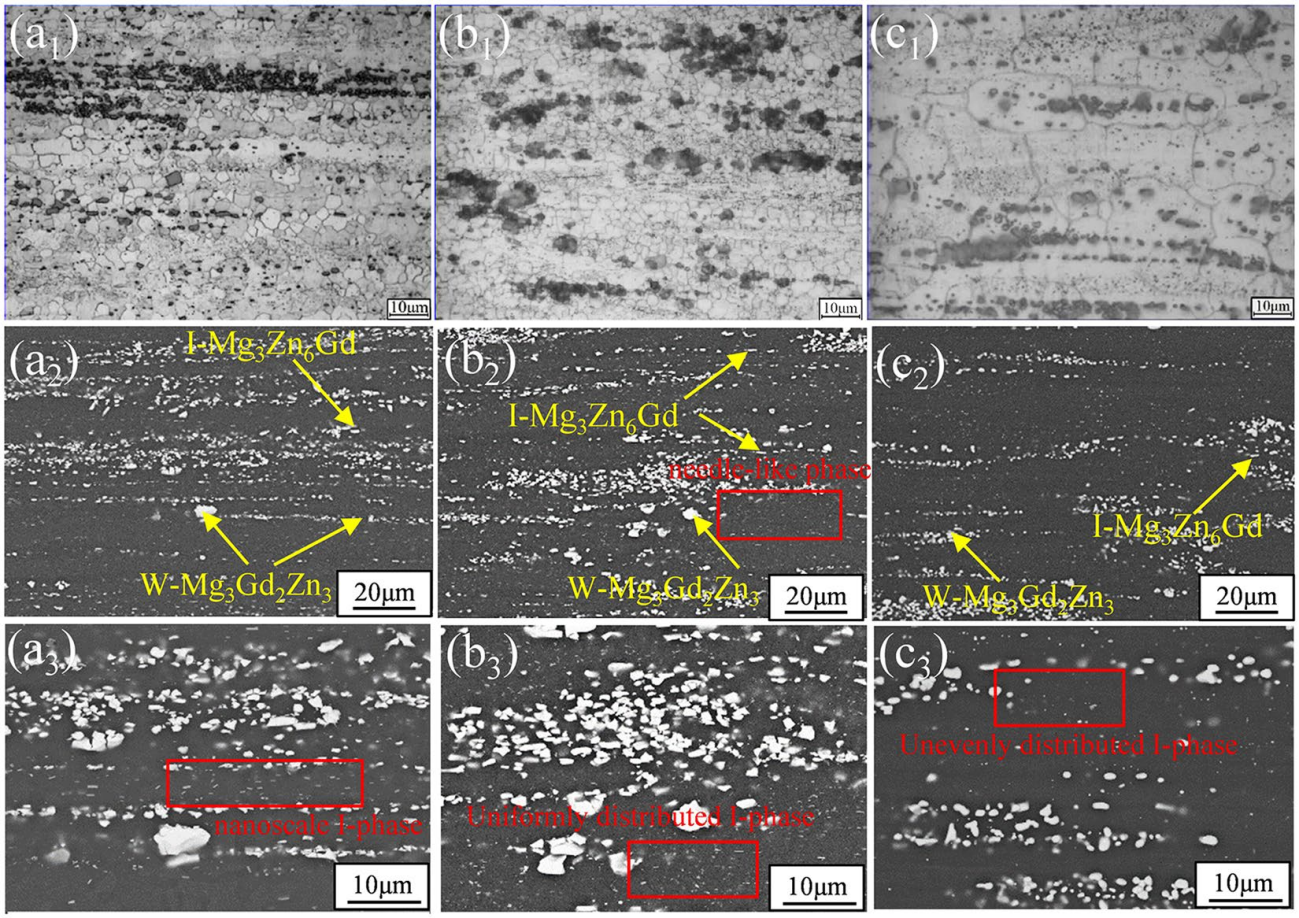
Optical microstructures and SEM micrographs of the GZ57K alloy: (**a**_**1**_), (**a**_**2**_) and (**a**_**3**_) as-extruded condition; (**b**_**1**_), (**b**_**2**_) and (**b**_**3**_) T5-treated condition; (**c**_**1**_), (**c**_**2**_) and (**c**_**3**_) T6-treated condition.

The optical microstructural features and the SEM micrographs of E, E + T5 and E + T6 alloys are shown in Fig. 6. After hot extrusion, the main bulk-like and strip-like secondary phases are zonal and elongated distributing along the extrusion direction. The initial I-phase is broken into uniformly distributed quasicrystal particles of 0.5–2 μm in size during extrusion, and these particles act as sources of dynamic recrystallization during hot extrusion, which could help to refine the grains. According to the report that aging at 180 °C had a beneficial effect on the mechanical properties of Mg-1.5Y-6Zn-0.5Zr (wt%) alloy due to the intensive precipitates^22^. Due to the 200 °C aging temperature in this work, these nanoscale particles could precipitate during T5 treatment. Also, the incomplete dynamic recrystallization (DRX) occurs in the as-extruded alloy and almost completely static recrystallization occurs in the E + T5 alloy (Fig. 6(a_1_,b_1_)). This is probably because the recrystallized grains are somewhat nucleation at the needle-like particles in the T5 condition, meanwhile, the nanoscale particles could also restrain the grain growth. For another, the shear stress generated during extrusion plays a key role in the elongated phases and grains. In the E + T6 alloy, the I-phase distributes unevenly in the magnesium matrix. To better confirm the compound constitutions, EDS is further employed. In Fig. 6(a_2_), chemical constituent of the strip-like phase manifests that the concentration of Zn and Gd are 15.08 at% and 2.47 at%, respectively. It indicates that the strip-like phase is I-Mg_3_Zn_6_Gd. EDS analysis of the small granular and irregular bulk phase is nearly Mg-27.13 at% Gd-42.39 at% Zn, which is close to W-Mg_3_Gd_2_Zn_3_ phase. Notably, the W-phase and I-phase morphologies are not changed during heat treatments. However, some fine needle-like phase appears in the E + T5 alloy.

**Figure 7:**
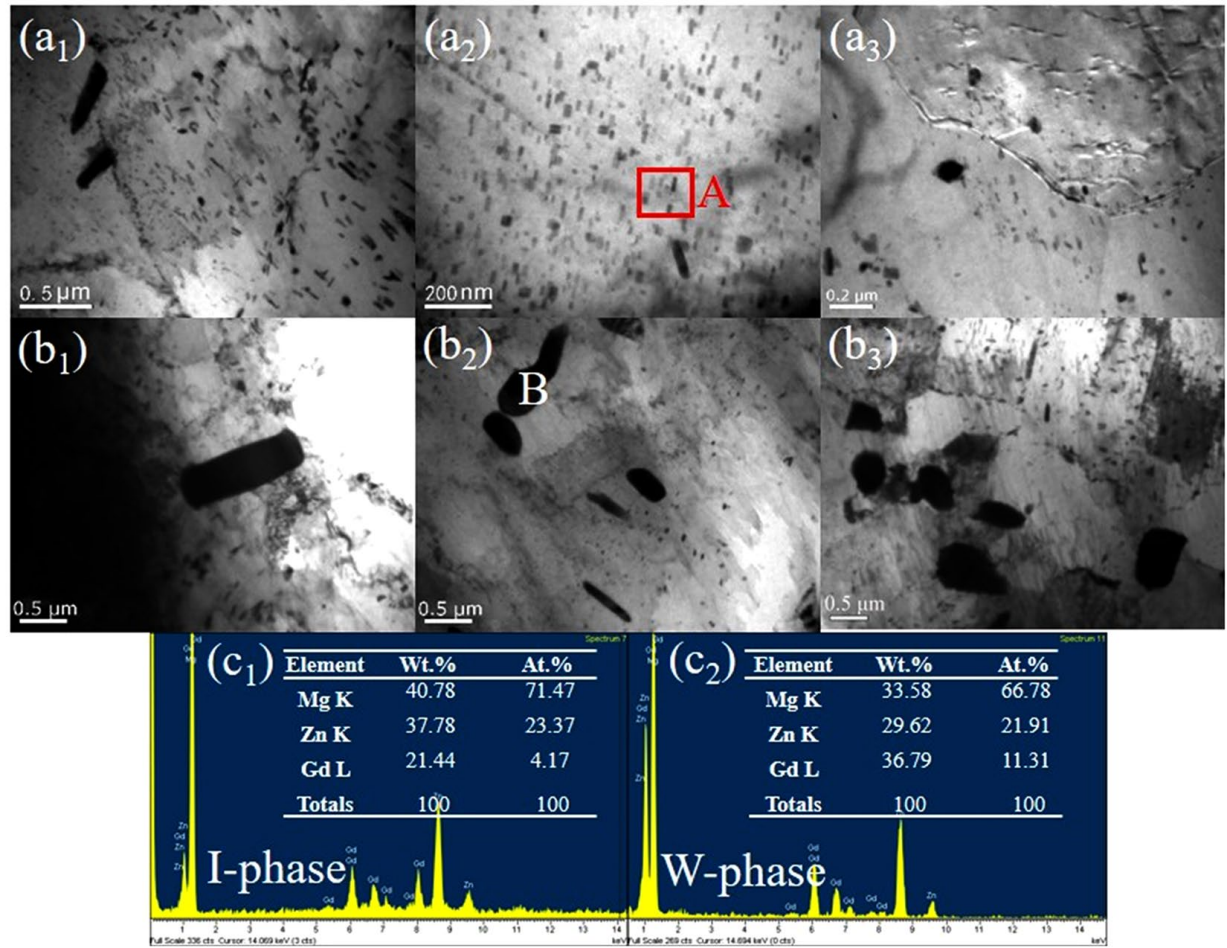
TEM images of the E + T5, E + T6 alloy: (**a**_**1**_), (**a**_**2**_) and (**a**_**3**_) E + T5; (**b**_**1**_), (**b**_**2**_) and (**b**_**3**_) E + T6; (**c**_**1**_) and (**c**_**2**_) EDS analysis of region A and point B.

For purpose of further confirming the morphologies and compositions of the phases observed under SEM, the magnified TEM microstructures and relevant EDS results of the second phases and the precipitated needle-like phases in the E + T5 and E + T6 alloys are shown in Fig. 7. The as-homogenized I-phase is crushed during the extrusion process, leading to the fine I-phase particles (0.5–2 μm) disperse in the matrix after T5 and T6 treatments, as shown in Fig. 7(a_1_,b_1_). A high density of fine needle-like nanoscale precipitations locates uniformly and compactly in the E + T5 alloy as shown in Fig. 7(a_1_,a_2_). In Fig. 7(a_3_), the grain boundaries (GBs) are pinned by the second phase. EDS test is conducted on region A as shown in Fig. 7(c_1_), and constituents of the nanoscale strip-like phases are Mg-21.37Zn-7.17Gd (at%), which is similar to the I-phase precipitated in the heat treatment process. Whilst, only a little bit of nanoscale I-phase precipitates in the E + T6 alloy. Because of the high solution treated temperature, parts of the W-phase become rounded after T6 treatment, Fig. 7(c_2_) reveals the specific composition of T6-treated W-phase. From Fig. 7(a_3_,b_3_), the precipitated nanoscale I-phase which located at the GBs could inhibit the grain growth during heat treatments and result in a decrease in grain sizes. However, it is shown in Fig. 7(b_3_) that the residual large W-phase and I-phase exist inside the grains but not along the GBs, so they would not effective for cavity nucleation as commonly believed. A majority of cavities are clearly relevant to the particles located in the GBs and at the particle/grain boundary interfaces^23–25^, this may be beneficial for this alloy to get super ductility.

**Figure 8:**
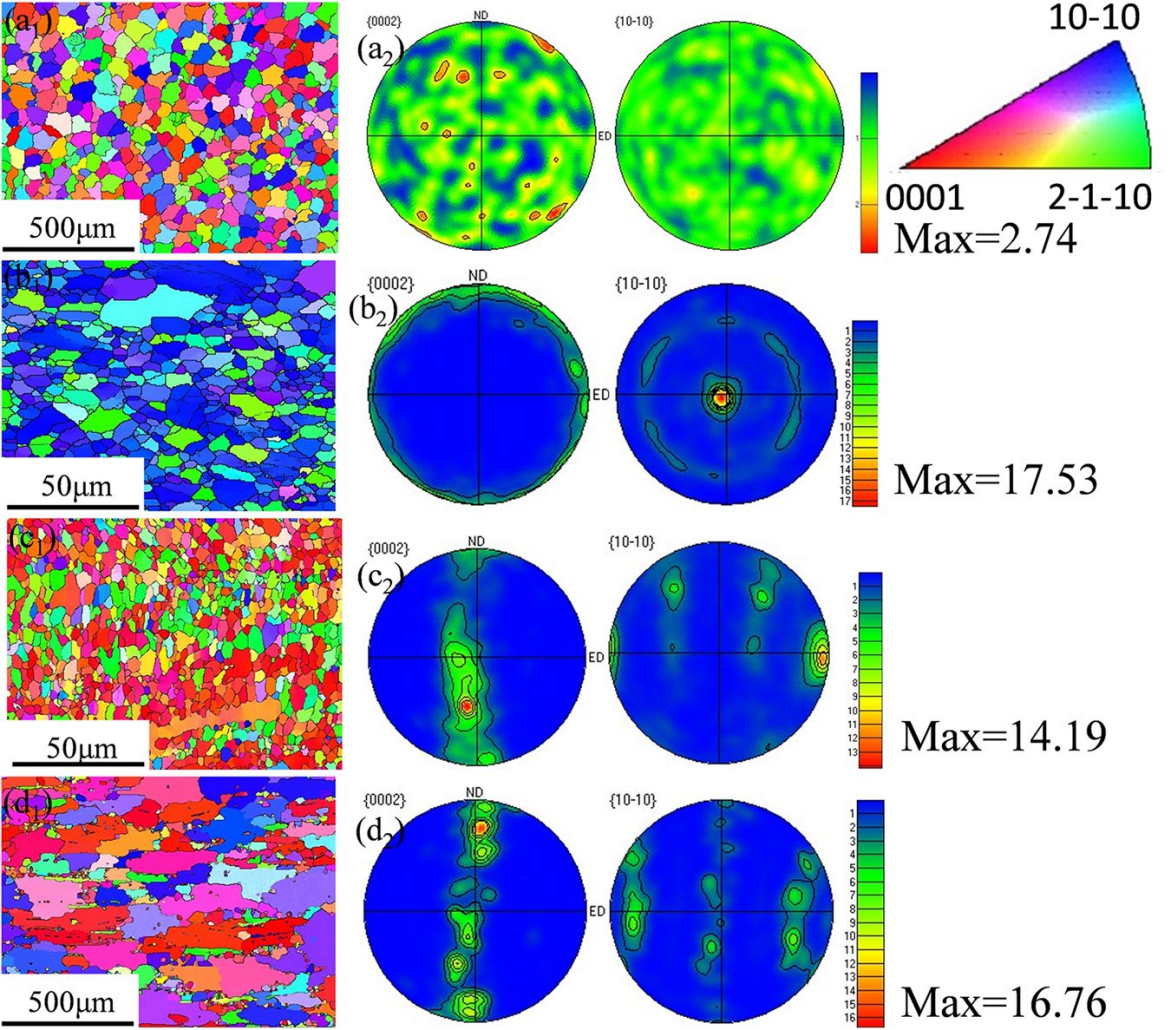
EBSD orientation maps and {0002} and {$10\bar{1}0$} pole figures for: (**a**) as-homogenized; (**b**) as-extruded; (**c**) E + T5; (**d**) E + T6 GZ57K alloy. (The extrusion direction is horizontal).

**Figure 9:**
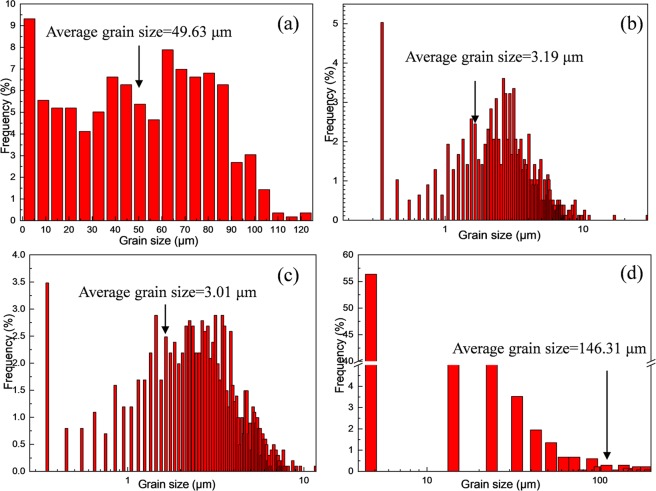
Grain size distributions corresponding to Fig. 8(a_1_–d_1_).

The orientation maps and pole figures before and after indirect extrusion are revealed by the EBSD maps as depicted in Fig. 8. Simultaneously, Fig. 9 shows the distributions of grain size corresponding to Fig. 8. In Figs 8(a_1_) and 9(a), at the homogenized condition, all of the grains are equiaxial and the average size of the grains is 49.63 μm. Nonetheless, in Figs 8(b–d) and 9(b–d) corresponding to E, E + T5 and E + T6 alloys, respectively, all the grains are stretched and distributing along the extrusion direction. The grain sizes decrease to 3.19 μm and 3.01 μm through extrusion and subsequent T5 aging treatment, respectively. The E + T5 alloy exhibits a nearly uniformly fine-grained structure (Fig. 8(c_1_)) with reasonably equiaxed shape. As compared with the extruded grain size, pronounced grain growth takes place and the average grain size is up to 146.31 μm after T6 treatment. The reason for the grain growth is the grain growth during the period of holding at 430 °C for 8 h before aging treatment. In the homogenized alloy (Fig. 8(a_2_)), the texture shows uniform orientation distributions, and the texture of the as-homogenized alloy is weak because the crystalline orientations are nearly random during the homogenization process. Therefore, no special role in the initial texture on the microstructure behavior during the indirect extrusion deformation and subsequent heat treatments. The extruded alloy exhibits strong basal and circular texture with a peak intensity more than 8 multiples of a random distribution (as-homogenized) with <10-10> axis of the matrix parallel to extrusion direction. The difference is related to the directional nucleation and selective growth during the dynamic recrystallization. The E + T5 alloy shows a typical <10-10> fiber texture, and the (0002) basal texture is uniformly distributed along the direction perpendicular to the extrusion. The texture produced by aging treatment is beneficial for tensile strength along ED. The peak intensity is also weakened after T5 annealing treatment. Firstly, the particle stimulated nucleation (PSN) of recrystallization is suggested as the main factor for weakening the texture for the alloys which have high content of alloying elements^26^. Secondly, the uniformed I-phase granules are local lattice rotations and effective for the large elongation in the alloys containing I-phase^27–29^. It can be seen that considerable amounts of coarse grains, in addition to some residual fine grains in the GBs (Fig. 8(d_1_)), which could be owing to the solution treatment and aging treatment. Meanwhile, the E + T6 alloy exhibits a slightly weakened texture compared with the E alloy (Fig. 8(d_2_,b_2_)). Since the alloy is subjected to solution treatment, there are less orientation changes compared with the initial condition. Meanwhile, the grain growth due to solute segregation could also slightly weaken the {0002} (basal) and the {$10\bar{1}0$} (prismatic) texture^30^.

### Microstructures after superplastic transformation

**Figure 10:**
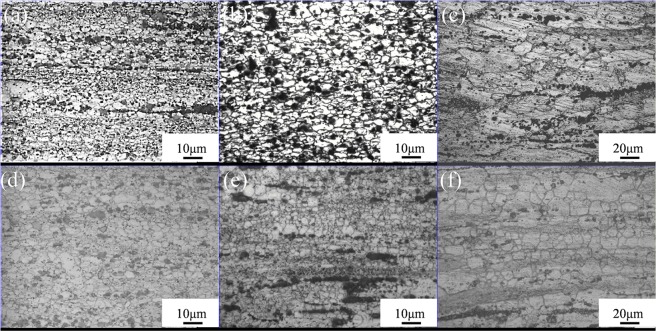
Optical microstructures of GZ57K alloy in uniform deformation and holding section after elevated temperature tensile experiment at a deformation temperature of 250 °C with different states: (**a**,**d**) E condition; (**b**,**e**) E + T5 condition; (**c**,**f**) E + T6 condition.

Figure 10 displays the optical micrographs of E, E + T5 and E + T6 alloys in uniform deformation and holding section after 250 °C superplastic deformation. All the microstructures consist of equiaxed grains, and the slight grain growth takes place compared with the undeformed alloys as shown in Fig. 6. The uniform deformation section grain sizes are shown in Table 1. This happened grain growth contains the static grain growth during the period of holding at 250 °C for 10 min before superplastic deformation and the deforming strengthened grain growth during tensile deformation. Meanwhile, the secondary phases still remain uniformly distributed in the matrix. Although the I-phase and W-phase have valid effects on inhibiting the grain growth, their effects are still restrained because of the low to medium temperatures and longer holding time. The grain shape in E + T5 is elongated along the tensile direction in the uniform deformation section but it remains equiaxed (Fig. 10(b)) and the elongation is 863%, indicating that GBS is the dominant deformation mechanism^31,32^.

**Table 1:** EBSD analysis results of the alloy specimens.

Processing	Average grain size before deformation (μm)	Average grain size after deformation (μm)	Number fraction of HAGBs (%)	Basal texture maximum intensity
As-homoginized	49.63		65	2.74
As-extruded	3.19	3.92	52	17.53
T5-treated	3.01	4.64	76	14.19
T6-treated	146.31	168.30	62	16.76

### Cavitation and fracture morphologies

**Figure 11:**
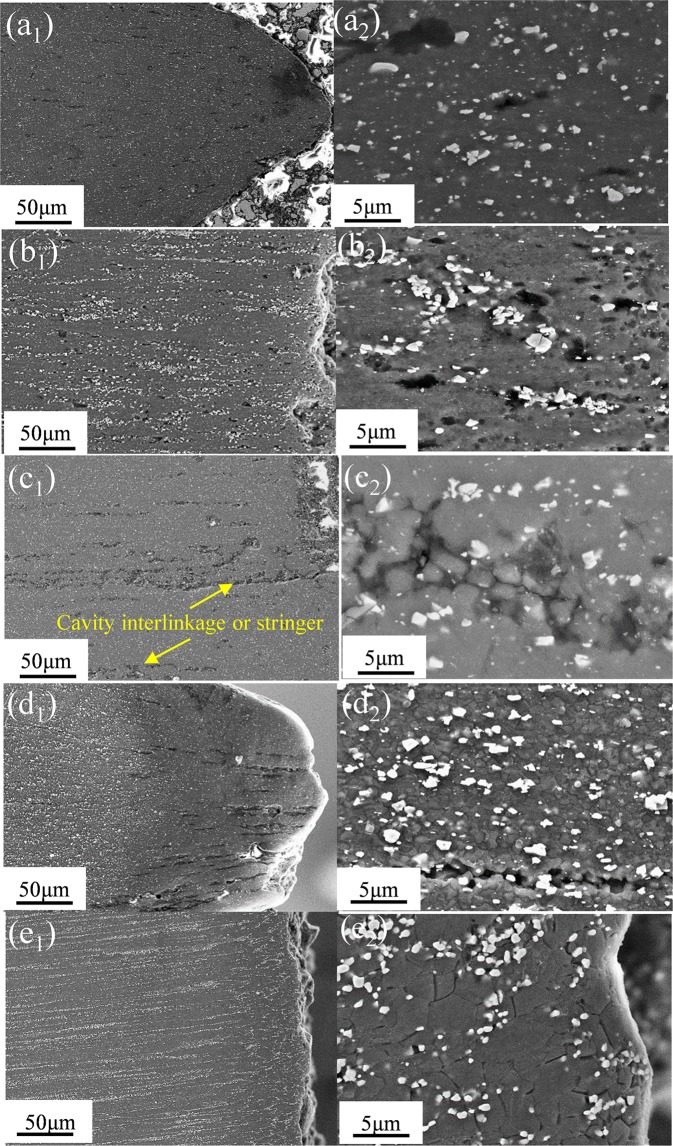
SEM images show the microscopic cavities after pulled to failure at different temperatures: (**a**_**1**_) and (**a**_**2**_) 150 °C; (**b**_**1**_) and (**b**_**2**_) 200 °C; (**c**_**1**_) and (**c**_**2**_) 250 °C of E + T5 alloy; (**d**_**1**_) and (**d**_**2**_) 250 °C of extruded alloy; (**e**_**1**_) and (**e**_**2**_) 250 °C of E + T6 alloy.

Figure 11 shows the microscopic cavities of E, E + T5 and E + T6 alloys after being stretched to failure at the strain rate of 1.67 × 10^−3^ s^−1^ with different temperatures. Without ambiguity, the average cavity diameters and the number of cavities in E + T5 alloy increase with the increasing tensile temperature. Cavities are visible at the three temperatures and these cavities are essentially rounded and elongated along the tensile axis. It is clearly observed from Fig. 11(a_2_,b_2_,c_2_) that many cavities nucleate at GBs, some cavities nucleate on the precipitates. These precipitates are the nanoscale I-phase. These particles could pin GBs and eliminate a large proportion grain growth at the elevated temperature. Whereas, on the other hand, these granules could also retard GBS, which is associated with cavities. This is probably because the dislocation piled-up increases at the triangular grain boundaries and the stress concentration causes some cavity nucleation as well as the cavity growth. In Fig. 11(c_1_), there are substantial cavity interlinkages near the fracture tip, and this interlinkage preferentially occurs parallel to the extruded axis. There are proofs for the alignment of these cavities into stringers parallel to the extruded axis at 250 °C condition. Inspection also shows that the interlinkage occurs through the sharp crack development along the GBs as shown in Fig. 11(c_2_). At the other temperatures, the number of cavities decreases probably because GBS decreases in these temperature conditions. Hence, the increased number of cavities does not reduce the plasticity of the alloy. Notably, the cavity interlinkage also appears in 250 °C specimen of the extruded alloy, and the cavity interlinkage develops into the cavity stringers as shown in Fig. 11(d_1_). It should be taken seriously that the interlinkage or stringer direction is consistent with the tensile direction, and the cavity could sustain a larger elongation subsequently before the final failure. If the interlinkage and stringer direction is perpendicular to the tensile direction, the cavity is tended to untimely failure. Results demonstrate that the cavity interlinkage direction is of great importance in achieving superior superplasticity. The number of the nanoscale I-phase in the E + T5 alloy is more than the E alloy as shown in Fig. 11(c_2_,d_2_). The sizes of second phase granules are crucial in the cavity initiation. Generally, equally distributed particles could inhibit synchronous grain growth and facilitate cavity nucleation. Fewer cavities could be seen in the E + T6 alloy, the GBs and the only small particles are nucleation sites for the cavities.

**Figure 12:**
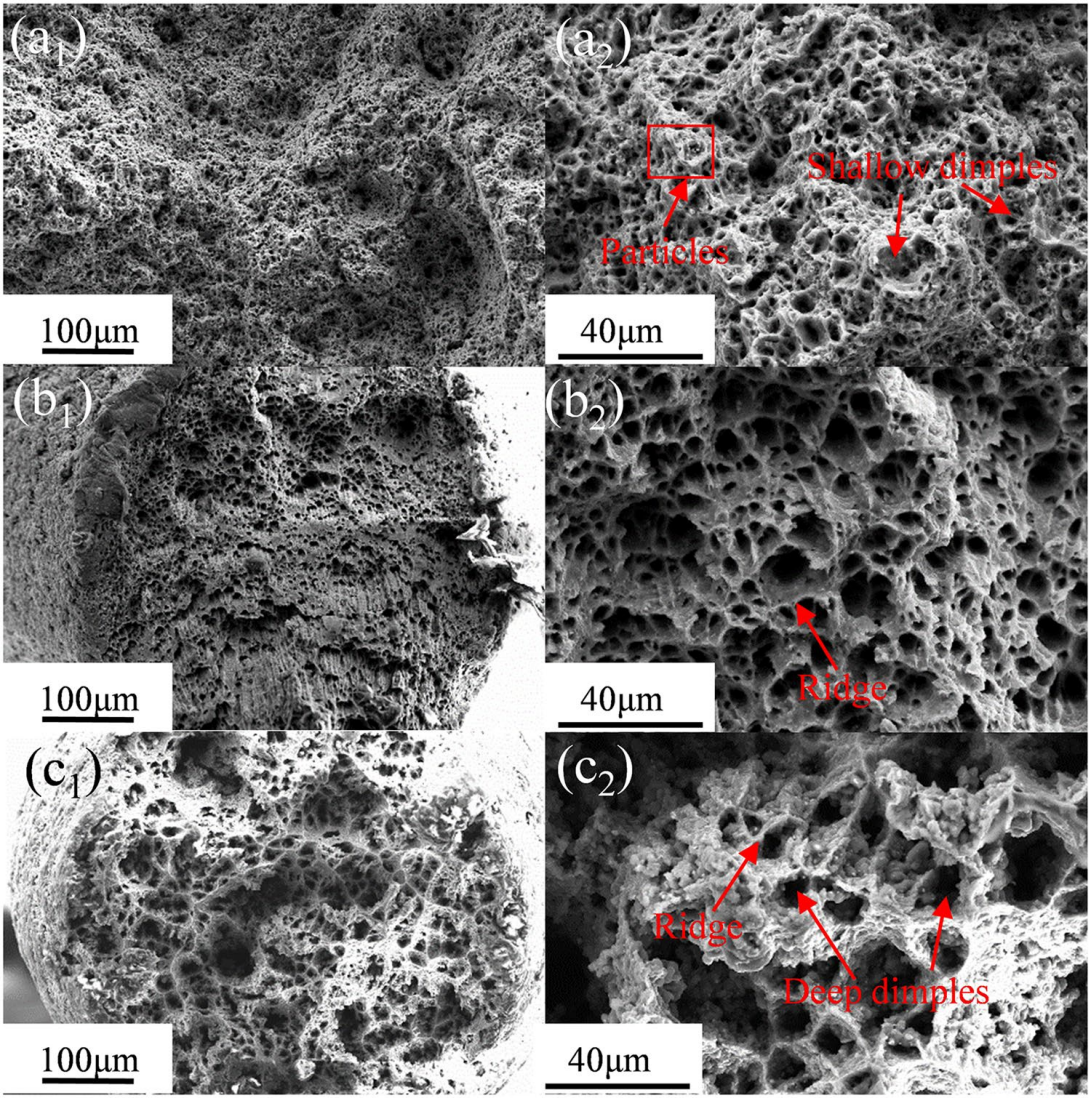
SEM images of fracture morphology of the E + T5 specimens that are tested at different temperatures: (**a**_**1**_) and (**a**_**2**_) 150 °C; (**b**_**1**_) and (**b**_**2**_) 200 °C; (**c**_**1**_) and (**c**_**2**_) 250 °C.

Figure 12 shows the fracture surfaces SEM images of the E + T5 alloy under the three tensile temperatures. It can be seen that many fine dimples appear in the three conditions and the average diameters of the dimples increase from 4.5 μm to 7 μm, and finally reach 9 μm at 250 °C. At the same time, the dimples in 250 °C are big and deep compared with the small and shallow ones in 150 °C as shown in Fig. 12(a_2_,b_2_,c_2_).

## Discussion

Very recently, it has been proved that the superplasticity forming of the I-phase containing Mg-Zn-RE (Gd, Y) alloys can be achieved at different conditions, the processing methods, tensile temperatures, the strain rates and elongations are summarized in Table 2^11,12,16,33–36^. To achieve the superplasticity of the alloys, complicated plastic deformation or high tensile temperatures are necessary. As is seen from Fig. 8(c) that after the conventional indirect extrusion and subsequent aging treatment, the microstructure is equiaxed grains and the average grain size is merely 3.01 μm. The obtained grain size and homogeneous microstructure is capable of reaching the standard grain size less than 10 μm so as to achieve superplasticity^37^. After tensile test, grains are slightly elongated but almost remained equiaxed and the microstructure is still fine grained with the grain sizes range from 5.5 to 8 μm. It indicated that there is no obvious grain growth happened during the superplastic deformation process and the microstructure is stable in the alloy. The thermally stable and equiaxed grains could provide the evidence of GBs occurrence. In addition, Figs 3 and 4 show the stable uniform deformation region of the E + T5 alloy at the strain rate of 1.67 × 10^−3^ s^−1^, the fracture also exhibits a diffuse necking. These characteristics demonstrate that this alloy is the typical fine-grained superplasticity. GBS occurs in the superplastic deformation process and the grain growth could take place during the deformation with no doubt. The secondary phases in the alloys are to inhibit the GBs or grain boundaries migration and so as to restrain the possible grain growth. The effective second phase and particle scales for fine grains are usually <1 μm^38^. The I-phase particles initially distribute along the extrusion direction (Fig. 6(a_2_)) and become randomly after T5 treatment. The particles do not coarsen. In addition, plenty of fine needle-like nanoscale I-phase granules are observed. The quasi-periodic lattice structure of I-phase owns stabilized I-phase particles/α-Mg phase interface^39^, due to the low lattice mismatching strain. Furthermore, the I-phase is thermostable in the α-Mg matrix. Because of the rigid coupling has comparatively low interfacial energy at the I-phase particles/α-Mg phase interface^40^, the tractive force for the I-phase granules coarsening could be low. These nanoscale granules could effectively make the microstructure stable and prevent grain growth so that superior superplasticity can be achieved. It is also noteworthy that the nanoscale I-phase precipitates are of high melting point so as to inhibit the grain growth at 250 °C effectively.

**Table 2:** Maximum elongation (Mechanical properties) of Mg-Zn-RE (Gd-Y)-Zr.

Alloy	Processing method	T/°C	Strain rate/s^−1^	δ/%	Ref.
Mg-1Zn-3Gd (wt%)	Extrusion + ECAP	400	3.3 × 10^−3^ s^−1^	800	^32^
Mg-4Gd-7Y-1Zn (wt%)	Extrusion	470	1.7 × 10^−4^ s^−1^	700	^33^
Mg-4.3Zn-0.7Y (wt%)	8 passes ECAP	350	1.5 × 10^−4^ s^−1^	600	^34^
Mg-4.3Zn-0.7Y (wt%)	Hot rolling	300	1 × 10^−3^ s^−1^	120	^16^
Mg-13Zn-1.55Y (wt%)	High speed rolling	250	1 × 10^−3^ s^−1^	1021	^35^
Mg-5.8Zn-1Y-0.48Zr (wt%)	Extrusion + ECAP	350	1.7 × 10^−3^ s^−1^	800	^11^
Mg-7.12Zn-1.2Y-0.84Zr (wt%)	Hot rolling + FSP	450	1 × 10^−2^ s^−1^	1110	^12^
Mg-7Zn-5Gd-0.6Zr (wt%)	Extrusion	250	1.67 × 10^−3^ s^−1^	863	this work

In the tensile deformation process, the dislocations pile up confronted with the nanoscale I-phase granules first, and the subsequent dislocations are hindered at the interface between I-phase and α-Mg phase. It is because these dislocations could not restrain the flow stress further increase but flow strain could be increased due to the Orowan relation. This mechanism explains the high ductility of alloys with nanoscale I-phase.

**Figure 13:**
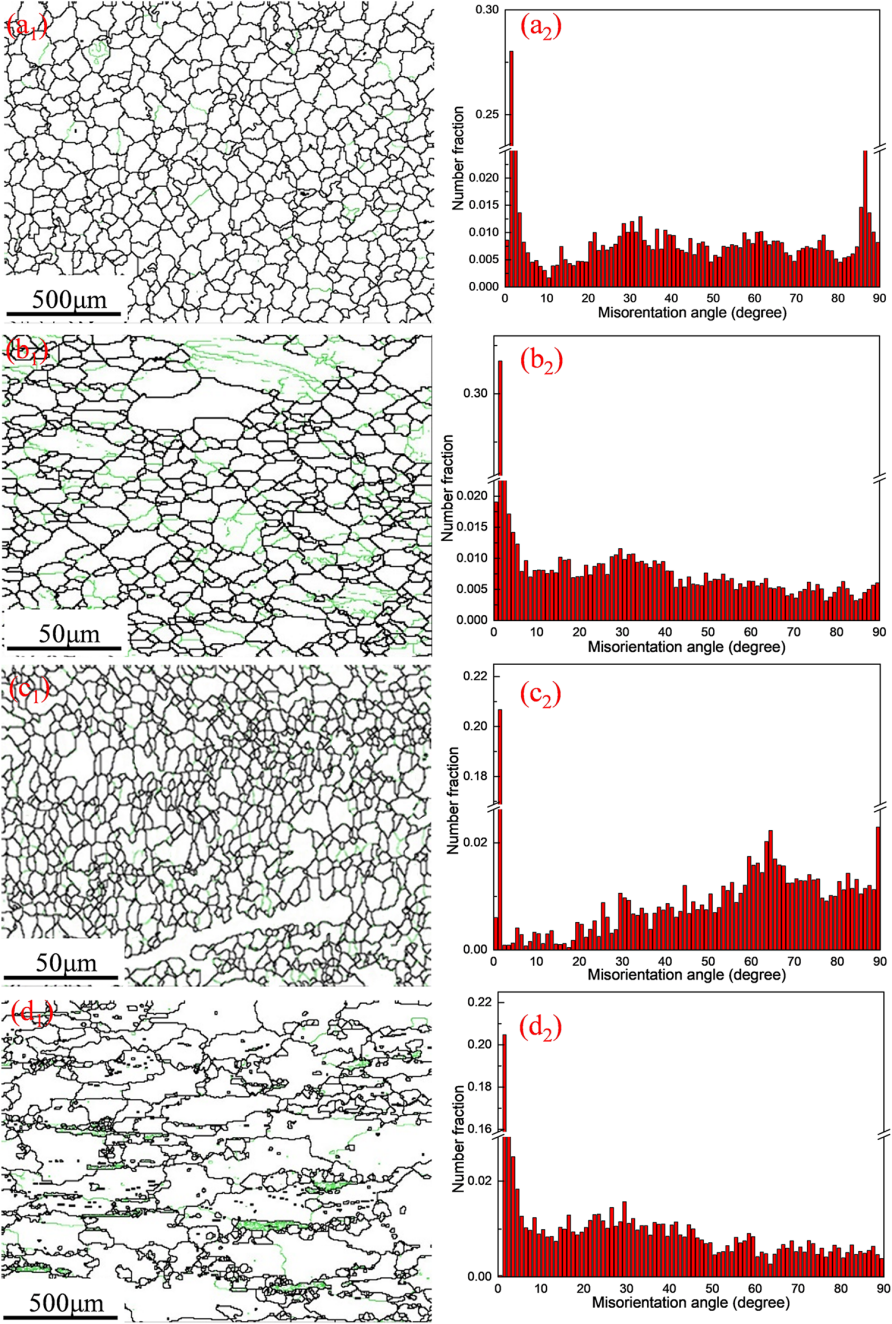
The EBSD orientation maps and number fractions of the misorientation angles of GZ57K alloy: (**a**_**1**_) and (**a**_**2**_) the as-homogenized; (**b**_**1**_) and (**b**_**2**_) the as-extruded; (**c**_**1**_) and (**c**_**2**_) T5-treated; (**d**_**1**_) and (**d**_**2**_) T6-treated GZ57K alloy. (The green lines are referred to low angle grain boundary).

**Figure 14:**
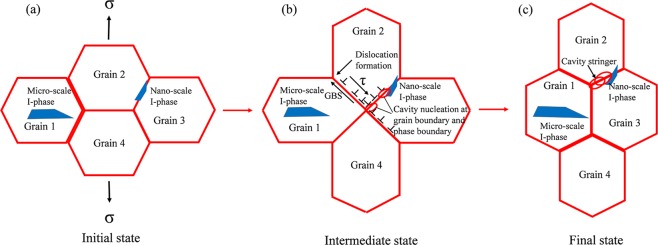
The schematic model of cavity nucleation and growth mechanism for the E + T5 alloy: (**a**) The initial state; (**b**) the intermediate state; (**c**) the final state.

As is known to all that the GBS is of great importance in the superplastic deformation process and researches manifest that GBS provides more than 50% of total strain in the superplastic deformation^41^. It is obvious that GBS would lead to the stress concentrations development at particles, triple points and cavities could nucleate when the stress concentrations are relieved incompletely. It is also apparent that the second phase granules at the GBs serve as the cavity nucleation points in aluminum alloys^42^. As shown in Fig. 13 and Table 1, there is an increasing number fraction of high angle boundary (HAGBs, θ ≥ 15°) after T5 treatment compared with the E and E + T6 alloys. The T5 treatment after extrusion results in the coarse grains are almost swallowed by the recrystallization process (Fig. 13(b_1_,c_1_)). The number fraction of HAGBs increases further. These high fractions of HAGBs demonstrate that high-angle boundaries are of most boundaries which can indirect manifest the GBS occurrence. Since the HAGBs own the high mobility under external stress, boundary sliding quickly occurs and superior superplasticity is easily to achieve. The owned high fraction of HAGBs coupled with the finer grain structure (3.01 μm) in the E + T5 alloy result in the superior *m*-values and GBS in the elevated temperature tensile testing^20^. GBS is generally accepted superplastic deformation mechanism in reasonable temperature and strain rate ranges. The microscopic GBS leads to large macroscopic strain and enhances the ductility. In Fig. 11(c_2_,e_2_), cavities originate preferentially at the GBs and I-phase particles/α-Mg phase, which is comparable to the Al-12Si-0.7Mg alloy^43^. The reason for the interface cavitation is that the phase and the soft α-Mg matrix own different abilities for boundary sliding. M.F. Ashby and R.A. Verrall proposed the A-V classical GBS coordinate deformation model^44^. In this model, four hexagonal grains as a unit will not deform and only the positions of the four grains change under the tensile stress, so the grains are still equiaxed after superplastic deformation. On account of this A-V classical model, the schematic model of the cavity nucleation and growth mechanism for the E + T5 experimental alloy is shown in Fig. 14. The GBS activates firstly and proceeds under the tensile strength, at the same time, the dislocations generate at the triple junction because the GBS is inhibited by the front grains and the stress concentration leads to the dislocations generation as shown in Fig. 14(b). The dislocations move down the grain boundary in the opposite direction of the GBS under the action of external shear stress τ and accumulate at the opposite triple junction. The stress concentration at the head of the piled-up dislocations could not be relaxed and the cavity forms when the piled-up stress exceeds the theory decohesion stress of the grain boundary or the second phase/α-Mg matrix. Along with the superplastic tensile process, extra cavities form and the original cavities grow to develop cavity stringers as shown in Figs 11(c_1_) and 14(c). The microstructures after the superplasticity exhibit approximately equiaxed grains which is in favor of the A-V classical deformation model as shown in Fig. 10.

## Conclusions

In present work, the lower-temperature superplastic behavior of E, E + T5 and E + T6 GZ57K alloy with I-phase is studied by tensile tests with the strain rate of 1.67 × 10^−3^ s^−1^ and temperatures range from 25 °C to 250 °C. Conclusions can be obtained as follows:

- The T5-treated GZ57K alloy exhibits the superior ductility and the optimum elongation of 863% is obtained at the strain rate of 1.67 × 10^−3^ s^−1^and the temperature of 250 °C. The elongation of the alloy during superplastic deformation increases with the increasing tensile temperature.
- The initial I-Mg_3_Zn_6_Gd phase and W-Mg_3_Gd_2_Zn_3_ phase are crushed into small granules during extrusion. A high density of nanoscale I-phase precipitates after T5 treatment. The E + T5 alloy shows weak basal texture intensity, a large number fraction of high angle boundary and a very finer grain size of 3.01 μm.
- During superplastic deformation, the nanoscale I-phase is slightly elongated and the microstructure is still equiaxed grains. Cavities nucleation at the GBs or nanoscale I-phase/α-Mg matrix boundaries and the cavity stringer formation leads to final fracture.
- The superplastic mechanism of the alloy is GBS accommodated by dislocation movement and static recrystallization.

## Data Availability

The authors declare that the materials and data are available and replicated without any controversy.
